# Getting Closer to an Underdiagnosed Disease: Hemosuccus Pancreaticus, a Rare Cause of Upper Gastrointestinal Bleeding

**DOI:** 10.7759/cureus.30837

**Published:** 2022-10-29

**Authors:** José D Cardona, Julián Alberto Beltrán Saavedra, Maria Rueda, Andrea Blanco, Luisa M Muñoz Quiroga, José M Hernandez Buelvas

**Affiliations:** 1 Radiology, University Hospital Fundación Santa Fé de Bogotá, Bogotá, COL; 2 Radiology, Instituto Nacional de Cancerología, Bogotá, COL; 3 Radiology, Fundación Santa Fe, Bogotá, COL; 4 Research, Universidad de La Sabana, Chia, COL

**Keywords:** minimally invasive interventional radiology, interventional radiology guided embolization, endovascular treatment, hemosuccus pancreaticus, upper gastrointestinal(ugi) bleeding

## Abstract

Hemosuccus pancreaticus is a rare cause of upper gastrointestinal hemorrhage. It is mainly produced by bleeding from a pseudoaneurysm that runs through the pancreatic duct and flows into the second portion of the duodenum. This article presents a case of a patient in the sixth decade of life with upper gastrointestinal bleeding who underwent a computed tomography (CT) scan of the abdomen finding a pseudoaneurysm of the gastroduodenal artery. Subsequently, angiography confirmed active bleeding from the pseudoaneurysm, requiring endovascular treatment by interventional radiology, which was successful with the resolution of the bleeding. In this article, our aim is to expand the information on this pathology and to promote the optimization of diagnostic tests for the timely treatment of this rare disease that is potentially life-threatening.

## Introduction

Hemosuccus pancreaticus (HP), also known as pseudohemobilia or wirsungorrhagia, is a life-threatening cause of upper gastrointestinal bleeding (UGI) that runs through the pancreatic duct [1-2]. First described in 1931 by Lower and Farrell, it was not until 1970 that Sandblom first coined the term after reporting three patients with bleeding pseudoaneurysms [3-4]. HP is one of the least frequent etiologies of UGI bleeding, with an estimated incidence of one in every 1,500 cases [5]. It is usually caused by the appearance of a pseudoaneurysm occurring in 3.5% to 10% of cases of pancreatitis. Rupture of a pseudoaneurysm is a relatively rare but life-threatening complication of chronic pancreatitis, happening in 6-8% of patients with pseudocysts and accounting for less than 1% of cases of upper gastrointestinal bleeding [3, 6, 7].

Other causes, such as tumors or vascular malformations, can be seen, with a higher incidence in males (7:1 ratio), and with a mean age of presentation between 50-60 years. Given its anatomical location, as well as its presentation with intermittent bleeding, HP cannot always be identified in esophagogastroduodenoscopy (EGD). The relevance of this condition lies in that it is a source of usually non-recognized GI hemorrhage that can be potentially fatal without an adequate diagnostic approach [8].

This article describes the case of a patient with a history of pancreatectomy for metastatic disease who presented upper gastrointestinal bleeding due to a pseudoaneurysm in the gastroduodenal artery and active bleeding confirmed by angiography. In addition, it highlights the importance of suspecting this pathology and the timely treatment.

## Case presentation

**Figure 1:**
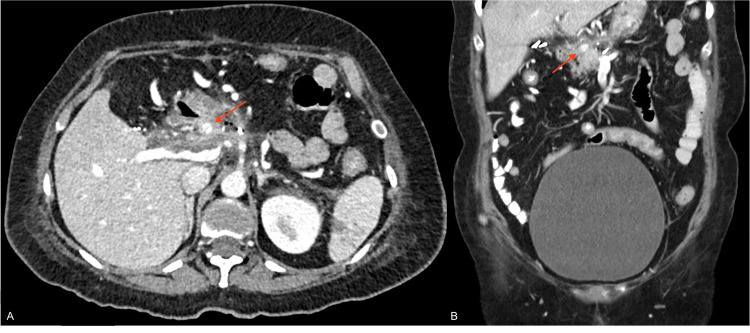
Contrast Enhanced CT scan images in axial (A) and Coronal (B) planes shows inflammatory changes in the surgical site of the partial pancreatectomy. Additionally, a redounded-like circumscribed enhancement is seen as compatible with a pseudoaneurysm (Arrows).

**Figure 2:**
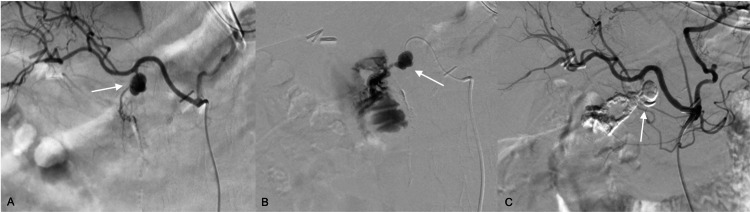
Angiographic images (A) show saccular dilatation corresponding to a pseudoaneurysm is documented as a function of the gastroduodenal artery. (B) Active bleeding in the duodenum is confirmed. (C) Control angiography showed adequate embolization using cyanoacrylate (histocryl) diluted with lipidol at a ratio of 1:2.5, demonstrating occlusion of the pseudoaneurysm and resolution of the hemorrhage.

A 67-year-old female presented to the emergency department with upper gastrointestinal tract bleeding. The patient had a history of kidney cancer with a right nephrectomy and had recently undergone a partial pancreatectomy and pancreaticogastrostomy because of pancreatic metastasis. At the emergency department, a contrast abdominal CT was ordered. Although the initial report only acknowledged the postsurgical changes with inflammatory findings next to the surgical site. A second look by the Interventional Radiologist suggested a highly attenuating image with iodine contrast inside, secondary to a pseudoaneurysm in the gastroduodenal artery (Figure 1A-1B). Initially, an EGD was planned, but after the latest radiological review, the patient was taken to angiography due to suspicion of HP. After the aortogram was performed, a pseudoaneurysm was confirmed demonstrating active bleeding towards the duodenum through the pancréatic duct (Figure 2A-2B). Based on the angiographic findings, an endovascular procedure was performed with adequate occlusion of the pseudoaneurysm with the resolution of the hemorrhage (Figure 2C).

## Discussion

Hemosuccus pancreaticus is one of the rarest forms of UGI bleeding. It occurs as a consequence of conditions related to anatomical disorders or irrigation abnormalities of the pancreas. The most common cause of bleeding is the rupture of a pseudoaneurysm, formed due to acute or chronic pancreatitis, especially from the splenic artery, followed by gastroduodenal and hepatic artery pseudoaneurysms [5,9]. Other etiologies related to HP are pancreatic malignancies, pseudocysts, and traumatic or iatrogenic injuries [10,11]. Clinical manifestations include epigastric pain associated with intermittent and progressive GI small-volume bleeding. Pain occurs due to the pressure exerted on the pancreatic duct with the secondary formation of a blood clot, which then is released from the pancreatic duct into the GI lumen, therefore presenting mainly with intermittent episodes of melena and, less frequently, hematemesis. Laboratory tests show nonspecific findings like iron deficiency anemia and also when pancreaticobiliary reflux occurs, hyperbilirubinemia is also described [5,7,12]. 

The complexity of the diagnosis of HP comes from its intermittent and insidious nature and its frequency of presentation. On average, the duration of symptoms until HP is diagnosed is more than 30 days [9]. EGD does not often detect the source of bleeding, and it is estimated that about 30% of bleedings coming from the papillae are identified through endoscopy, though it does help to exclude other GI bleeding etiologies. Given the non-pathognomonic clinical course, imaging studies are essential to achieve a better approach to this pathology. Ultrasonography helps in identifying pancreatic pseudocysts or near-pancreatic aneurysms. Contrast-enhanced computed tomography with angiography represents an excellent diagnostic tool, revealing structural abnormalities of the pancreas, such as features of chronic pancreatitis, pseudocysts, as well as its surrounding vasculature [13], and leads to diagnosis in 90% of cases [11]. Interventional therapies are the first-line option given in hemodynamically stable patients [14], with high success and reduced mortality, which is why selective arteriography is the gold standard for diagnosis and treatment, with a sensitivity of 96% and a success rate higher than 70%, and even 88% in some recent studies [9, 10]. Arteriography helps identify the culprit artery and details the affected anatomy [15]. 

Surgery is reserved for hemodynamically unstable patients in whom a previous less invasive procedure had failed or it is not feasible, though a mortality rate of 28% to 56% has been described in these cases [14]. If left untreated, HP is almost always fatal, with mortality rates greater than 90% [14], which is why prompt diagnosis and treatment are imperative in these cases.

## Conclusions

Inflammatory changes in the post-surgical pancreas (post-surgical pancreatitis), can cause catastrophic damage to the surrounding tissue. Blood vessels, many times overlooked, are not the exception. It is important to adequately interpret imaging in these patients and pay specific attention to not miss important and potentially life-threatening diagnoses. 

Hemosuccus pancreaticus is a rare cause of gastrointestinal bleeding and is many times a challenging diagnosis for inexperienced eyes. A prompt and correct diagnosis can direct the patient to the appropriate treatment given that a delay in definitive management could be life-threatening. A percutaneous procedure by an interventional radiologist is the first-line treatment given that the endoscopic approach will not be able to identify the source of bleeding. Timely recognition and adequate management could potentially reduce mortality and morbidity in patients presenting with HP.
